# Osteogenically committed hUCMSCs-derived exosomes promote the recovery of critical-sized bone defects with enhanced osteogenic properties

**DOI:** 10.1063/5.0159740

**Published:** 2024-02-06

**Authors:** Shuyi Li, Qiong Rong, Yang Zhou, Yuejuan Che, Ziming Ye, Junfang Liu, Jinheng Wang, Miao Zhou

**Affiliations:** 1 Guangdong Cardiovascular Institute, Guangdong Provincial People's Hospital, Guangdong Academy of Medical Sciences, Guangzhou 510080, China; 2 Department of Stomatology, Guangdong Provincial People's Hospital (Guangdong Academy of Medical Sciences), Southern Medical University, Guangzhou 510080, China; 3 Department of Oral and Maxillofacial Surgery, Affiliated Stomatology Hospital of Guangzhou Medical University, Guangdong Engineering Research Center of Oral Restoration and Reconstruction, Guangzhou Key Laboratory of Basic and Applied Research of Oral Regenerative Medicine, Guangzhou 510182, China; 4 Department of Stomatology, The First People's Hospital of Yunnan Province, The Affiliated Hospital of Kunming University of Science and Technology, Kunming 650032, China; 5 Zhongshan City People's Hospital, Affiliated Zhongshan Hospital of Sun Yat-sen University, Zhongshan 528400, China; 6 Department of Anesthesia, Sun Yat-sen Memorial Hospital, Sun Yat-sen University, Guangzhou 510120, China; 7 Guangzhou Municipal and Guangdong Provincial Key Laboratory of Protein Modification and Degradation, State Key Laboratory of Respiratory Disease, School of Basic Medical Sciences, Guangzhou Medical University, Guangzhou 511436, China

## Abstract

Low viability of seed cells and the concern about biosafety restrict the application of cell-based tissue-engineered bone (TEB). Exosomes that bear similar bioactivities to donor cells display strong stability and low immunogenicity. Human umbilical cord mesenchymal stem cells-derived exosomes (hUCMSCs-Exos) show therapeutic efficacy in various diseases. However, little is known whether hUCMSCs-Exos can be used to construct TEB to repair bone defects. Herein, PM-Exos and OM-Exos were separately harvested from hUCMSCs which were cultured in proliferation medium (PM) or osteogenic induction medium (OM). A series of *in-vitro* studies were performed to evaluate the bioactivities of human bone marrow mesenchymal stem cells (hBMSCs) when co-cultured with PM-Exos or OM-Exos. Differential microRNAs (miRNAs) between PM-Exos and OM-Exos were sequenced and analyzed. Furthermore, PM-Exos and OM-Exos were incorporated in 3D printed tricalcium phosphate scaffolds to build TEBs for the repair of critical-sized calvarial bone defects in rats. Results showed that PM-Exos and OM-Exos bore similar morphology and size. They expressed representative surface markers of exosomes and could be internalized by hBMSCs to promote cellular migration and proliferation. OM-Exos outweighed PM-Exos in accelerating the osteogenic differentiation of hBMSCs, which might be attributed to the differentially expressed miRNAs. Furthermore, OM-Exos sustainably released from the scaffolds, and the resultant TEB showed a better reparative outcome than that of the PM-Exos group. Our study found that exosomes isolated from osteogenically committed hUCMSCs prominently facilitated the osteogenic differentiation of hBMSCs. TEB grafts functionalized by OM-Exos bear a promising application potential for the repair of large bone defects.

## I. INTRODUCTION

Bone defects caused by tumor resection, trauma, or congenital deformities in the craniomaxillofacial region severely affect the health status and facial esthetics of patients.^1,2^ Autografts are considered as the “gold standard” to repair bone defects because they bear excellent biocompatibility, osteoconductivity, osteoinductivity, and osteogenesis-related cells.^3,4^ However, several shortcomings of autografts, such as the secondary operation area and limited bone volume restrict the clinical application. As alternatives, allografts,^5^ xenografts,^6^ and artificial bone grafts^7^ that bear similar structures and compositions to bone tissue, have been widely used in the clinic. Nevertheless, most of these bone grafts have no intrinsic osteoinductive elements. In addition, their geometries do not match the anatomy of bone defects, which need to be trimmed during surgery, resulting in an expensive and time-consuming process.^8^ Up to now, the repair of large bone defects is still challenging for clinicians.

With the rapid development of regenerative medicine and bone tissue engineering over the past decades, tissue-engineered bone (TEB) built on seed cells and biomaterial scaffolds shows a promising application potential due to favorable biocompatibility, excellent pro-osteogenic properties and customized morphology.^9^ Mesenchymal stem cells (MSCs) are the most widely used seed cells in preclinical and clinical studies on various bone diseases.^10^ They can be isolated from abundant tissues, such as bone marrow and umbilical cord.^11,12^ Compared with human bone marrow MSCs (hBMSCs), human umbilical cord MSCs (hUCMSCs) are atraumatically isolated from discarded umbilical cords. They have a higher cell yield and rapid proliferation property than hBMSCs.^13^ In addition, hUCMSCs can still maintain cell morphology and high telomerase activity without senescence signs after 15 passages.^14,15^ HUCMSCs have been regarded as promising seed cells for bone tissue engineering.^16^ Our previous study demonstrated that osteogenically- and angiogenically committed hUCMSCs co-cultured at the ratio of 3:1 possessed much better osteogenic ability than pure osteogenically committed hUCMSCs. In addition, they showed excellent restorative outcomes when combined with 3D printed tricalcium phosphate (TCP) scaffold to repair calvarial defects in rats.^17^ Moreover, the secreted factors of hUCMSCs can also promote the osteogenic differentiation of MSCs and new bone regeneration.^18^ Nevertheless, the usage of MSCs is restricted by the storage condition and cell senescence during *in-vitro* expansion.^19^ In addition, low cell viability and the potential of immunological rejection severely affect the final therapeutic efficacy when cell-based TEB are directly applied to promote bone regeneration.^20^

Exosomes (Exos) are nano-sized (range of 40–160 nm) extracellular vesicles of endosomal origin and can be secreted by almost all cell types.^21^ Exos carry a complex cargo of biological molecules, such as proteins, lipids, mRNAs, microRNAs (miRNAs), and long non-coding RNAs, thus playing a pivotal role in mediating intercellular communication.^21,22^ Among them, miRNA is crucial to regulate both the physiological and pathological processes through mRNA degradation or translational blockage.^23^ Studies show that miRNAs, such as let-7a, miR-199b, miR-218, and miR-375 in MSCs-derived Exos participate in and regulate bone metabolism.^24,25^ Compared with MSCs, Exos are advantageous in low immunogenicity, easy storage, strong stability, enhanced efficacy, and biological safety, which have been extensively studied in promoting bone regeneration.^26,27^ For example, Exos from dental pulp stem cells (DPSCs) facilitate the osteogenic differentiation of BMSCs.^28^ Exos secreted by adipose tissue-derived MSCs can promote the regeneration of alveolar bone.^29^ Up to now, limited studies have focused on how to obtain hUCMSCs-derived Exos of predictable and favorable efficacy in promoting the repair of bone defects and elucidating the underlying mechanisms.

In this study, we screened an optimal culture condition to harvest hUCMSCs-derived Exos with pro-osteogenic properties and explored the miRNA secretome. Furthermore, based on our previous studies on 3D printed customized TCP scaffolds, we incorporated Exos with pro-osteogenic properties into TCP scaffolds to construct a bioactive TEB to repair the calvarial bone defects of rats. This study would provide an application paradigm for the harvest of functional Exos and their usage in repairing large bone defects.

## II. RESULTS

### A. Osteogenic differentiation capacity of hUCMSCs

**FIG. 1.**
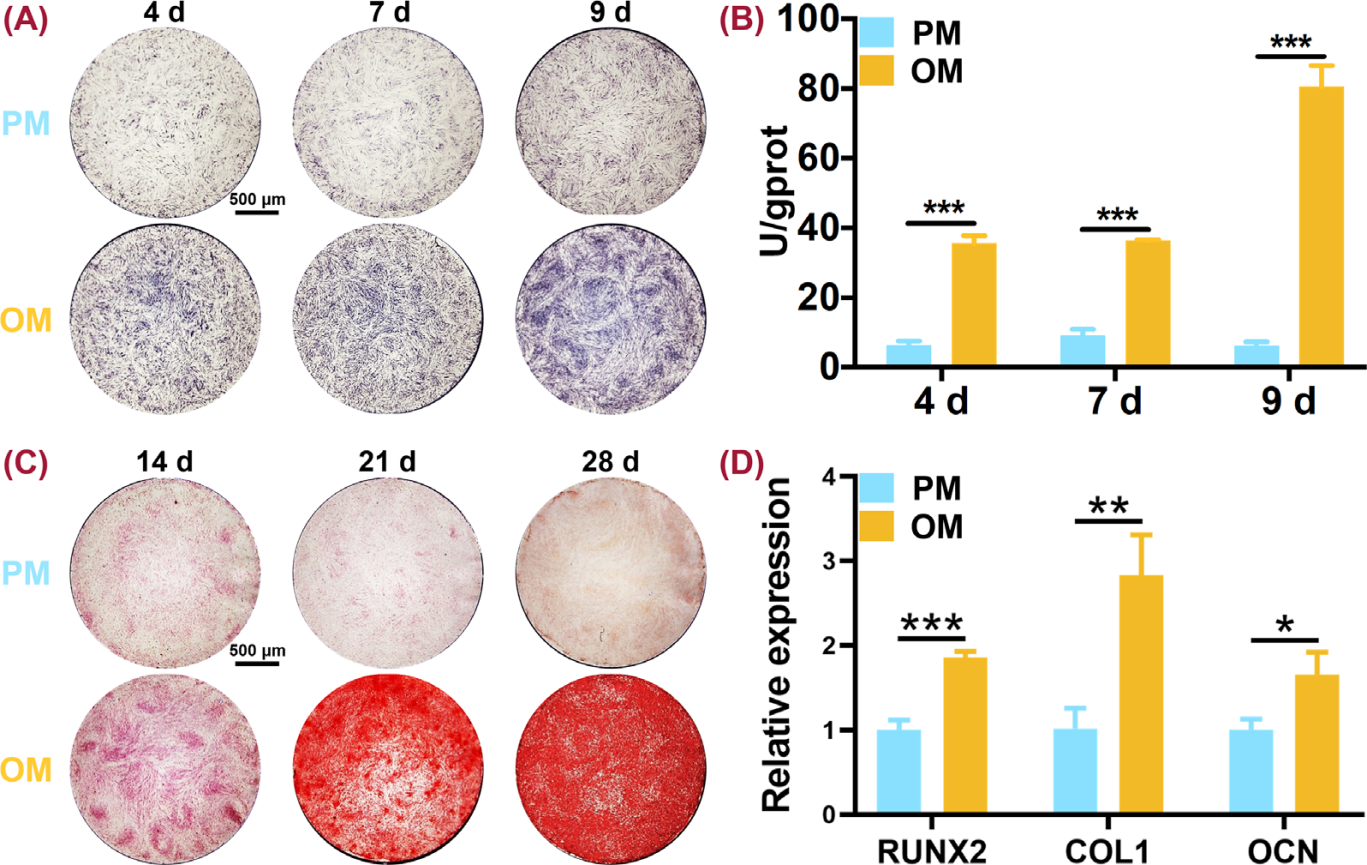
Osteogenic differentiation of hUCMSCs. (a) ALP staining of hUCMSCs and osteogenically committed hUCMSCs for 4, 7, and 9 days. (b) ALP quantitative analysis. (c) ARS staining of hUCMSCs and osteogenically committed hUCMSCs for 14, 21, and 28 days. More red-stained calcium nodules were observed in OM on days 14, 21, and 28. (d) qRT-PCR analysis of osteogenesis-related genes (RUNX2, COL1, and OCN) expression after 9 days. PM: proliferation medium; OM: osteogenic induction medium; ALP: alkaline phosphatase; ARS: Alizarin red S. ^*^P < 0.05; ^**^P < 0.01; and ^***^P < 0.001.

In order to examine the osteogenic differentiation capacity of hUCMSCs, the qualitative and quantitative analysis of ALP activity, osteogenesis-related genes expression as well as the formation of mineralized nodules were performed. After 4, 7, and 9 days of osteogenic induction, ALP staining of hUCMSCs in OM became purple, darker than that in PM, with the darkest color being observed on the ninth day [Fig. 1(a)]. The quantitative examination of ALP activity was consistent with ALP staining [Fig. 1(b)]. The ALP activity of hUCMSCs in OM increased significantly during the observed period compared with that of hUCMSCs in PM (*P* < 0.001) and reached the highest level on the ninth day, which was about twice as much as that on the fourth and seventh day. In addition, ARS staining showed an increase in calcium nodules when hUCMSCs were cultured in OM from day 14 to 28 while no calcium nodules were found in PM [Fig. 1(c)]. Moreover, osteogenically committed hUCMSCs showed significantly higher expression levels of RUNX2, COL1, and OCN than hUCMSCs (*P* < 0.05) [Fig. 1(d)].

**FIG. 2.**
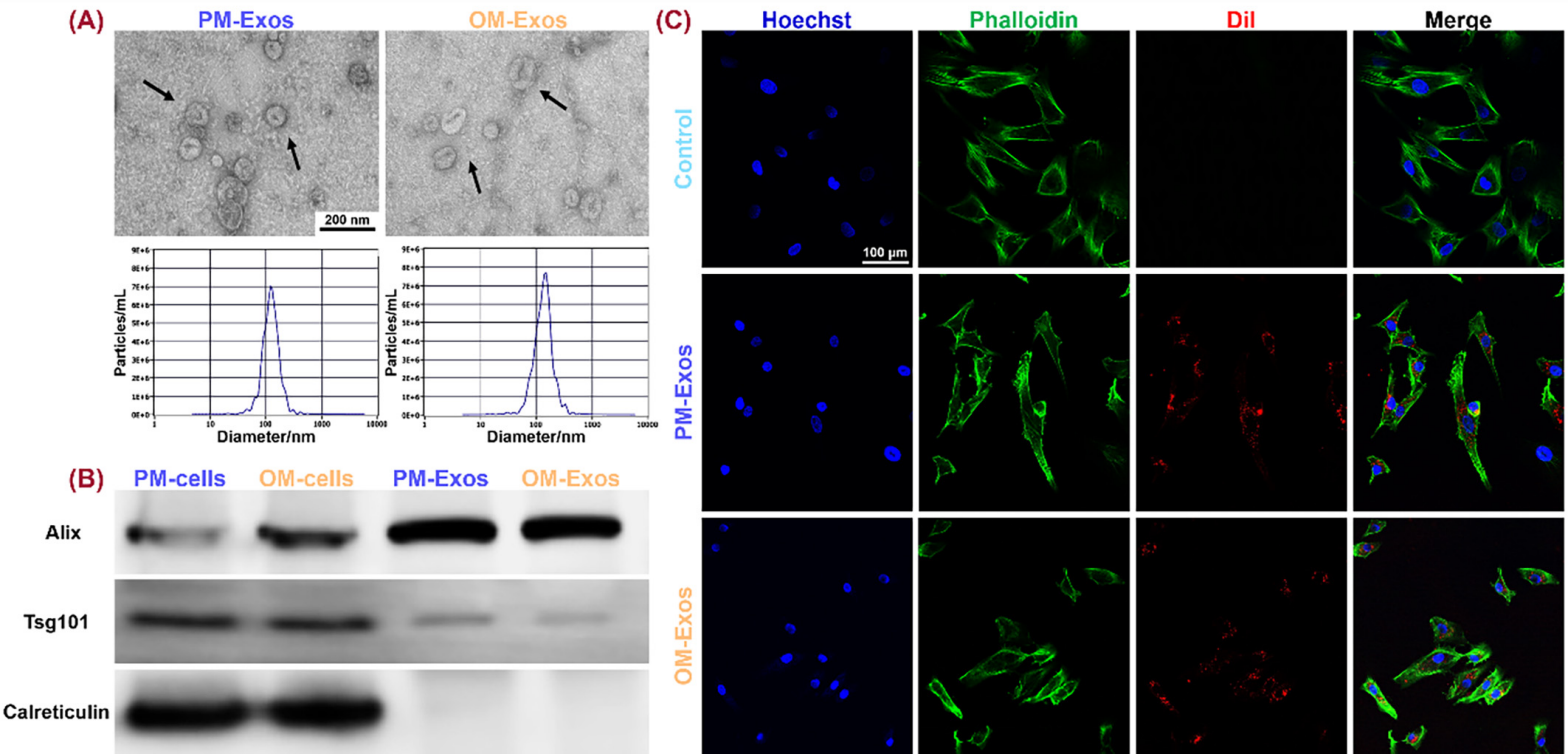
Identification of hUCMSCs-derived PM-Exos and OM-Exos and their internalization by hBMSCs. (a) TEM and NTA analysis of PM-Exos and OM-Exos. (b) Exosomal positive and negative markers in PM-Exos, OM-Exos, and the respective donor cells, i.e., PM-cells and OM-cells, tested by WB. (c) Internalization of Exos after being co-cultured with hBMSCs for 4 h. Hoechst-stained nuclei were blue, phalloidin-stained cytoskeleton presented green and Dil-labeled Exos showed red under CLSM.

### B. Identification of hUCMSCs-derived Exos and their internalization by hBMSCs

Both PM-Exos and OM-Exos displayed “cup-shaped” and “tea saucer-shaped” bilayer membrane structures shown by TEM [Fig. 2(a)]. NTA demonstrated that the average particle diameters of PM-Exos and OM-Exos were 130.3 ± 49.3 and 143.5 ± 59.6 nm, respectively, with no significant difference (*P* > 0.05) [Fig. 2(b)]. Both PM-Exos and OM-Exos possessed positive markers of Exos, such as Alix and TSG101, while didn't express Calreticulin which was only detected in the donor cells [Fig. 2(b)].

**FIG. 3.**
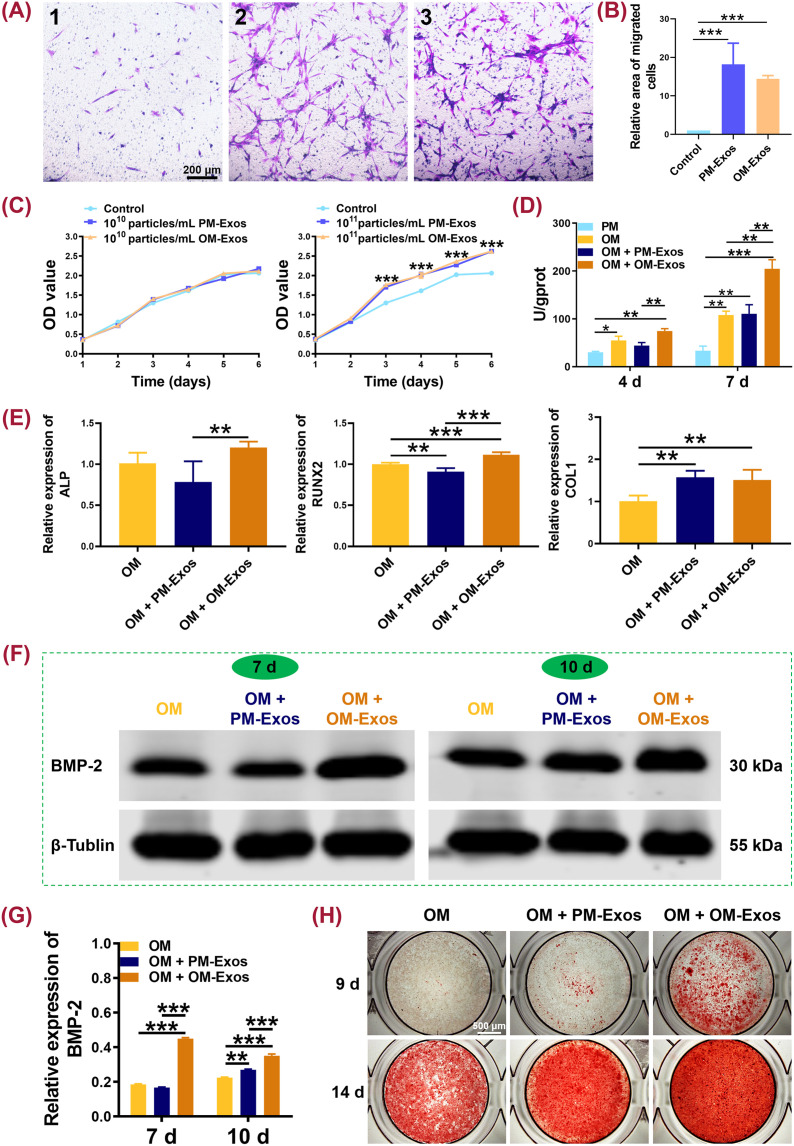
The effects of hUCMSCs-derived PM-Exos and OM-Exos on the migration, proliferation and osteogenic differentiation of hBMSCs. (a) The Transwell chemotaxis assay of hUCMSCs-derived PM-Exos and OM-Exos. (1) control; (2) PM-Exos; and (3) OM-Exos. (b) Relative area of migrated cells compared with the control group. (c) CCK-8 assay to evaluate the proliferation of hBMSCs when being treated with different concentrations of PM-Exos and OM-Exos from day 1 to day 6. (d) Quantitative analysis of ALP activity of hBMSCs on days 4 and 7. (e) qRT-PCR analysis of osteogenesis-related genes expression. (f) and (g) WB assay of BMP-2 expression in hBMSCs and its quantitative analysis on days 7 and 10. (h) ARS staining of hBMSCs on days 9 and 14. ^*^P < 0.05; ^**^P < 0.01; and ^***^P < 0.001.

CLSM revealed that after incubating with Hoechst and phalloidin staining solution, hBMSCs bore dark blue-stained nuclei and green-stained cytoskeleton [Fig. 2(c)]. The Exos labeled by Dil were red, which overlapped with the green cytoskeleton in hBMSCs, indicating that both PM-Exos and OM-Exos could be internalized by hBMSCs after 4 h of co-culture. As PBS could not be labeled by Dil, the red color could not be found in hBMSCs in the control group.

### C. The effects of PM-Exos and OM-Exos on the bioactivities of hBMSCs

Transwell chemotaxis assay revealed that both PM-Exos and OM-Exos significantly promoted the migration of hBMSCs with a larger cellular area than that of the control group (*P* < 0.001) [Figs. 3(a) and 3(b)]. In the CCK-8 assay, when both PM-Exos and OM-Exos were at the concentration of 10^10^ particles/ml, the proliferation rates of hBMSCs were similar to that of the control group from day 1 to day 6 [Fig. 3(c)]. However, when the concentration increased to 10^11^ particles/ml, the proliferation rate was significantly promoted on the third, fourth, fifth, and sixth days (*P* < 0.001). There was no significant difference between the PM-Exos group and OM-Exos group (*P* > 0.05).

**FIG. 4.**
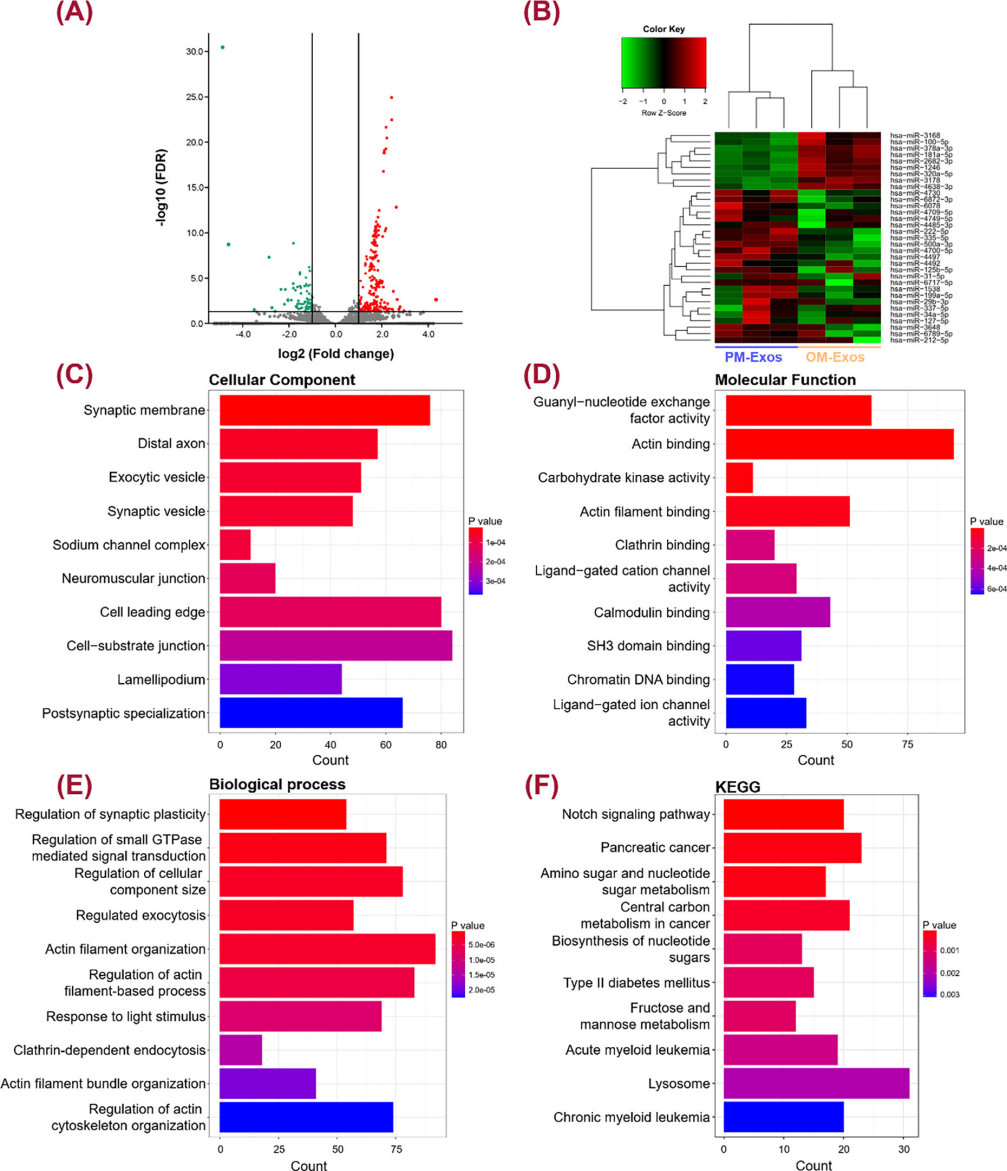
MiRNA sequencing of hUCMSCs-derived PM-Exos and OM-Exos. (a) Volcano plots of the differentially expressed small RNAs in PM-Exos and OM-Exos. The black dots represented small RNAs with no significant difference, the green dots for down-regulated small RNAs and the red dots for the up-regulated ones of statistical significance. (b) Heatmap of different miRNA profiles between PM-Exos and OM-Exos. (c)–(f) The target genes of the top 10 miRNAs were enriched in GO cellular component, molecular function, biological processes, and KEGG.

To further examine the pro-osteogenic activities of both PM-Exos and OM-Exos, ALP activity, qRT-PCR, WB, and ARS staining assays were adopted. The expression of ALP protein increased from day 4 to day 7 in all the studied groups [Fig. 3(d)]. On day 4, OM + OM-Exos could significantly enhance the expression level of ALP in hBMSCs (*P* < 0.01) compared with those in PM and OM + PM-Exos. There was no significant difference between OM + OM-Exos and OM groups (*P* = 0.05) as well as OM + PM-Exos and PM groups (*P* > 0.05). ALP activities of OM + OM-Exos were increased by 1.68 times than those of OM + PM-Exos and 1.35 times than those of OM on day 4. On day 7, the ALP activity of OM + OM-Exos was significantly higher than those of PM, OM, and OM + PM-Exos (*P* < 0.01). In addition, compared with PM, OM + PM-Exos significantly improved the ALP activity of BMSCs (*P* < 0.01). Specifically, ALP activities of OM + OM-Exos were increased by about 1.90 times than those of OM + PM-Exos and OM on day 7 [Fig. 3(d)]. The ALP gene expression of OM + OM-Exos was also higher than that of OM + PM-Exos (*P* < 0.01) [Fig. 3(e)]. In addition, the expression levels of RUNX2 and COL1 in OM + OM-Exos were significantly higher than those in OM (*P* < 0.01). Compared with OM + PM-Exos, the expression of RUNX2 significantly increased in OM + OM-Exos (*P* < 0.01). There was no significant difference between OM + PM-Exos and OM + OM-Exos referring to the expression of COL1. Moreover, the protein expression level of BMP-2 in OM + OM-Exos was higher than those in OM and OM + PM-Exos on both days 7 and 10 (*P* < 0.001) [Figs. 3(f) and 3(g)]. OM + PM-Exos also displayed a higher expression level of BMP-2 on day 10 than OM (*P* < 0.01).

As the final osteogenic differentiation marker, the formation of calcium nodules dramatically increased in OM + OM-Exos compared with those in OM and OM + PM-Exos on days 9 and 14. On day 9, a large number of calcium nodules formed in OM + OM-Exos while only a few scattered in OM and OM + PM-Exos. On day 14, the bottom of the well was occupied by calcium nodules in both OM + PM-Exos and OM + OM-Exos. The color of red-stained nodules in OM + OM-Exos was darker than that in OM + PM-Exos. Only patches of calcium nodules appeared in OM [Fig. 3(h)].

### D. Differentially expressed miRNAs in PM-Exos and OM-Exos

MiRNA sequencing on total RNAs extracted from Exos was applied to comprehensively evaluate the differentially expressed miRNAs between PM-Exos and OM-Exos. Volcano plots showed a total of 311 small RNAs were differentially expressed, of which 222 were found to be significantly up-regulated (red dots) and 89 down-regulated (green dots) in OM-Exos relative to PM-Exos. The black dots showed no significantly expressed small RNAs [Fig. 4(a)]. 33 miRNAs with significant differences (fold change > 2 and FDR < 0.05) in the volcano plot were selected, normalized (row Z-score), and drawn into a heat map [Fig. 4(b)]. Compared with PM-Exos, the expression levels of miRNAs, such as miR-181a-5p and miR-100-5p were upregulated, while miRNAs, such as miR-4700-5p, miR-212-5p, and miR-222-5p were downregulated. Bioinformatics analysis showed that both miR-4700-5p and miR-212-5p possessed specific binding sites in the 3′ UTR of OCN [supplementary material Fig. 1(a)]. Green-stained hBMSCs were detected by the fluorescence microscope, indicating both the mimic and inhibitor FAM-NC entered the cells [supplementary material Fig. 1(b)]. After being transfected by miR-4700-5p mimic, hBMSCs displayed down-regulated expression of OCN (*P* < 0.05). A similar phenomenon was observed when hBMSCs were transfected by miR-212-5p mimic. On the contrary, both miR-4700-5p inhibitor and miR-212-5p inhibitor dramatically enhanced the expression of OCN (*P* < 0.01) [supplementary material Fig. 1(c)].

**FIG. 5.**
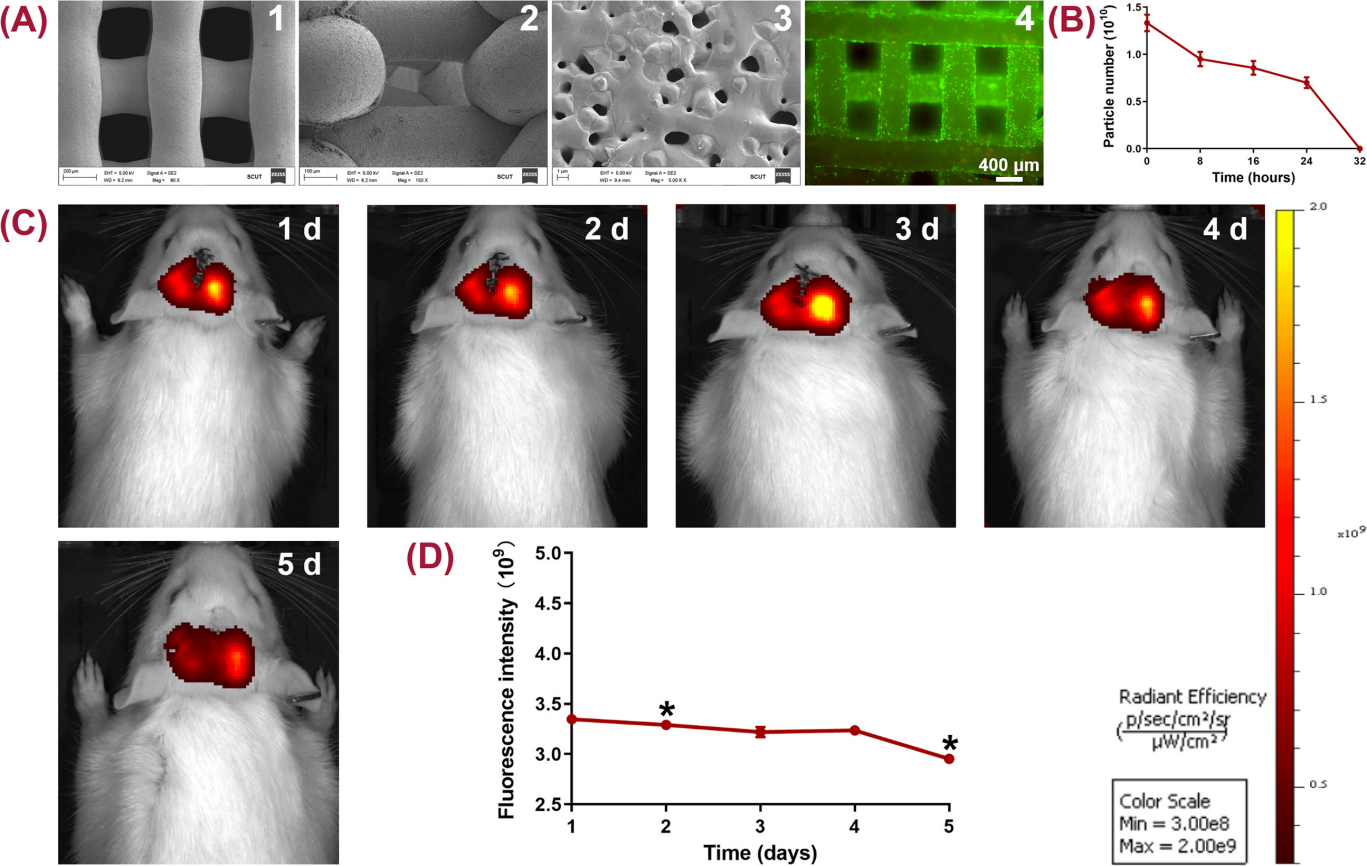
Characterization of 3D printed TCP scaffolds and the release kinetics of OM-Exos both *in vitro* and *in vivo*. (a) Hierarchically structured TCP scaffolds observed by SEM and cytocompatibility analysis. (a-1) Regular struts with a porous structure; (a-2) a lateral view of interconnected pores; (a-3) microporous and nanoporous structures of TCP; and (a-4) live/dead staining. (b) Particle number of OM-Exos released from the scaffolds when being incubated in PBS for 32 h. (c) and (d) *In vivo* imaging examination to detect the distribution and fluorescence intensity of DiR-labeled OM-Exos from day 1 to 5. ^*^P < 0.05.

GO analysis revealed that the upregulated miRNA were functionally enriched in “synaptic membrane,” “distal axon,” and “exocytic vesicle” in the cellular component, “actin binding,” “actin filament binding” and “guanyl-nucleotide exchange factor activity” in the molecular function, “actin filament organization,” “regulation of cellular component size,” and “regulation of small GTPase mediated signal transduction” in the biological process. In addition, the KEGG pathway enrichment was dominated in “pancreatic cancer,” “central carbon metabolism in cancer,” “Notch signaling pathway” and “amino sugar and nucleotide sugar metabolism” pathways [Figs. 4(c)–(f)].

### E. Characterization of the 3D printed TCP scaffolds

SEM showed that the 3D printed round scaffold consisted of regular struts with uniform pores [Fig. 5(a-1)]. The pores were highly interconnected from the lateral view [Fig. 5(a-2)]. At 5.0 K magnification, the scaffolds bore a rough surface with microporous and nanoporous structures [Fig. 5(a-3)]. Live/dead staining assay showed green-stained viable cells on the porous scaffolds, with few red-stained dead cells after 3 days [Fig. 5(a-4)].

**FIG. 6.**
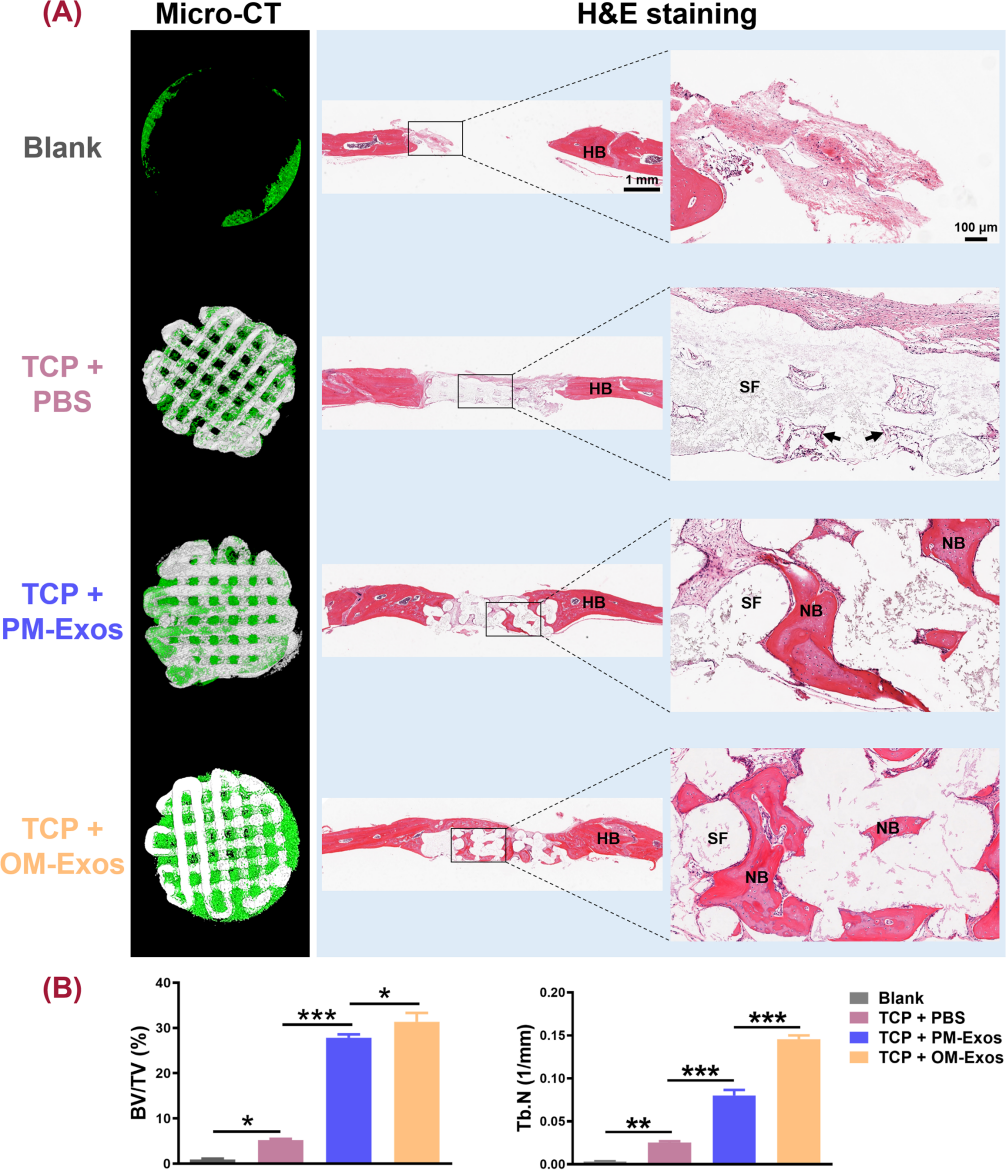
Rehabilitation of the calvarial bone defects by TEB. (a) Micro-CT and histological analysis. For micro-CT, green-stained tissue represented the newly formed bone, while the white stood for the 3D printed TCP scaffolds. For histological analysis, a large amount of red-stained homogeneous mature bone could be detected in TCP + OM-Exos group. The black arrows stand for osteoid. HB: host bone; SF: scaffold; NB: new bone. (b) Quantitative analysis of BV/TV and Tb.N. BV/TV: bone volume/total volume; Tb.N: bone trabecular number. ^*^P < 0.05; ^**^P < 0.01; and ^***^P < 0.001.

### F. Release kinetics of OM-Exos

NTA could only detect OM-Exos particles in the PBS medium within the initial 24 h, of which the particle number constantly reduced from (1.33 ± 0.15) × 10^10^ to (0.87 ± 0.13) × 10^10^. No particles were detected at 32 h, indicating that the residual number of OM-Exos in the TCP scaffold might be too small to be detected by NTA [Fig. 5(b)].

### G. Rehabilitation of calvarial bone defects

All the rats recovered soundly after the operation. *In-vivo* imaging of small animals was applied to validate the retention of OM-Exos, which showed that the red fluorescence maintained in the range of 2.95 ± 0.01 (× 10^9^) to 3.35 ± 0.03 (× 10^9^) at the defect area from day 1 to day 5 [Figs. 5(c) and 5(d)]. The statistical difference could be detected on day 2 as compared with that on day 1, of which the fluorescence intensity decreased from 3.35 ± 0.03 (× 10^9^) to 3.29 ± 0.03 (× 10^9^) (*P* < 0.05) [Fig. 5(d)]. The fluorescence intensity maintained from day 3 to day 4 and decreased from 3.24 ± 0.09 (× 10^9^) on day 4 to 2.95 ± 0.01 (× 10^9^) on day 5 (*P* < 0.05).

Micro-CT examination showed minor new bone formed around the defect in the blank group. A small amount of regenerated bone was detected along the pores and struts in TCP + PBS. When incorporating PM-Exos instead of PBS, much more new bone grew into the pores along the struts while almost all the pores were filled with newly formed bone in the TCP + OM-Exos group, even the surfaces of the scaffolds were covered by new bone [Fig. 6(a)]. Statistical analysis showed that both BV/TV and Tb.N were the highest in TCP + OM-Exos (31.40% ± 4.30%; 0.15 ± 0.01), significantly different from those in blank (0.96% ± 0.34%; 0.003 ± 0.001), TCP + PBS (5.18% ± 0.69%; 0.03 ± 0.004) and TCP + PM-Exos (27.85% ± 1.63%; 0.08 ± 0.01) (*P* < 0.05). In addition, the BV/TV and Tb.N of TCP + PM-Exos were also significantly higher than those in blank and TCP + PBS (*P* < 0.05) [Fig. 6(b)].

**FIG. 7.**
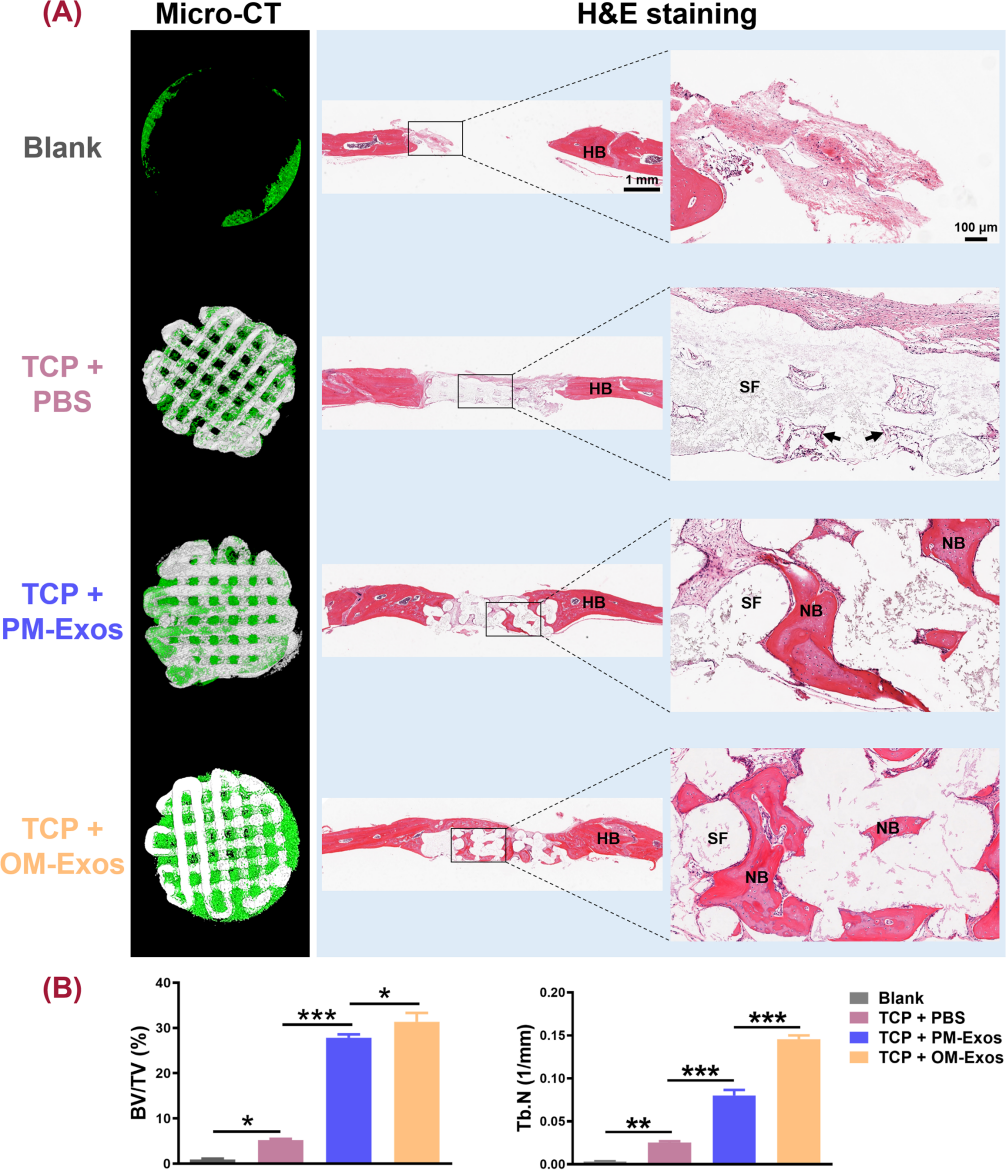
Rehabilitation of the calvarial bone defects by TEB. (a) Micro-CT and histological analysis. For micro-CT, green-stained tissue represented the newly formed bone, while the white stood for the 3D printed TCP scaffolds. For histological analysis, a large amount of red-stained homogeneous mature bone could be detected in TCP + OM-Exos group. The black arrows stand for osteoid. HB: host bone; SF: scaffold; NB: new bone. (b) Quantitative analysis of BV/TV and Tb.N. BV/TV: bone volume/total volume; Tb.N: bone trabecular number. ^*^P < 0.05; ^**^P < 0.01; and ^***^P < 0.001.

H&E staining was applied to further evaluate the restorative outcome. Consistently, only minor newly formed bone was detected in the periphery of calvarial defects in the blank group while the defect area was filled with connective tissues. In the TCP + PBS group, the scaffold was mainly surrounded by connective tissues. Only a small amount of osteoid generated at the margin of pores in TCP scaffolds. Compared with TCP + PM-Exos, more mature bone tissue regenerated along the porous structures in TCP + OM-Exos [Fig. 6(a)].

## III. DISCUSSION

Due to the predictable efficacy and biological safety, the application of stem cell-derived Exos in regenerative medicine has attracted more and more attention. To provide a novel strategy to fabricate bioactive TEB for the repair of craniomaxillofacial bone defects, our study, for the first time, exploited and compared two Exos from hUCMSCs, i.e., OM-Exos (from osteogenically committed hUCMSCs) and PM-Exos (from hUCMSCs). Extensive *in-vitro* studies of transwell chemotaxis assay and CCK-8 revealed that both OM-Exos and PM-Exos significantly promoted the proliferation and migration of hBMSCs. ALP, qRT-PCR, WB, and ARS showed that OM-Exos were superior in promoting the osteogenic differentiation of hBMSCs compared with PM-Exos. MiRNA sequencing displayed that the differentially expressed miRNAs might be responsible for the pro-osteogenic property. Furthermore, when incorporated in 3D printed TCP scaffolds, OM-Exos significantly accelerated new bone formation and the final restorative outcome compared with PM-Exos, indicating OM-Exos were promising in promoting the osteogenic outcome when being used to repair bone defects.

Compared with the traditional usage of MSCs, their secreted Exos do not express the major histocompatibility complex proteins (e.g., MHC-I and MHC-II), thus avoiding allogeneic rejection.^30^ Exos can deliver a variety of biologically active molecules (e.g., nucleic acid, proteins, and lipids) to the recipient tissue and their lipid bilayer membrane structure can protect these molecules from degradation during transportation. Exos can even cross biological barriers (e.g., blood-brain barrier) due to their relatively small size. Therefore, Exos possess favorable biocompatibility, bioactivity, phenotypic stability, and targeting ability, which confer Exos an important role in regenerative medicine. For example, Exos from BMSCs hold great regenerative potential while Exos from hUCMSCs are prominent in the repair of tissue damage.^31^ PM-Exos from hUCMSCs can enhance fracture healing by HIF-1α-mediated angiogenesis and Wnt-mediated new bone formation.^32,33^ Nevertheless, pure PM-Exos may not be potent enough to repair large bone defects. Therefore, we isolated OM-Exos and compared them with the traditional isolated PM-Exos. Our *in-vitro* results showed that OM-Exos and PM-Exos shared similar morphology, size distribution, and surface markers. They could also be internalized by hBMSCs to promote cell proliferation and migration, which is consistent with other studies.^34,35^ Specifically, when at the concentration of 10^10^ particles/ml, PM-Exos and OM-Exos bore similar functions to the control group in promoting the proliferation of hBMSCs; when the concentration increased to 10^11^ particles/ml, both PM-Exos and OM-Exos could significantly enhance the proliferation of hBMSCs. Therefore, the lowest dosage of Exos that could influence the proliferation of hBMSCs was 10^11^ particles/ml.

A series of examinations were also conducted to validate the pro-osteogenic properties of Exos. As an early marker of osteoblast phenotype, ALP activities continuously increase from pre-osteoblasts to mature osteoblasts.^36^ The expression of ALP gene of OM-Exos was 1.20 and 1.54 times higher than those of control and PM-Exos on day 4. Meanwhile, the expression of ALP protein in OM-Exos was 1.68 and 1.85 times higher than that in PM-Exos on days 4 and 7. In addition, as a crucial transcription factor during the osteogenic differentiation of MSCs, the expression of RUNX2 was 1.23 times higher than that of PM-Exos. Both OM-Exos and PM-Exos bore similar expression of COL1, about 1.5 times higher than the control. Furthermore, BMP-2 has been widely recognized as a key factor in differentiating MSCs into osteoblasts during bone regeneration, which can bind to the receptors of target cells to activate Smad and non-Smad signaling pathways.^37^ The expression of BMP-2 in OM-Exos was 2.69 and 1.30 times higher than those of PM-Exos on days 7 and 10. Furthermore, as the final osteogenic marker during osteogenesis, more calcium nodules were detected in OM-Exos than in PM-Exos both on days 9 and 14. Therefore, all the *in-vitro* results validated that OM-Exos were more potent than PM-Exos in accelerating osteogenesis.

Apart from the addition of pro-osteogenic factors, tissue-engineered scaffolds that bear excellent mechanical support and porous structure to resist tissue collapse and facilitate the migration, attachment, and proliferation of MSCs are essential during the repair of large bone defects. With similar organic compositions to native bone tissue, tunable degradation rate, and mechanical strength, TCP is a promising bioactive material to repair bone defects.^38^ To satisfy various clinical scenarios of large bone defects, our group has developed a series of customized TCP scaffolds with hierarchically porous structures by robocasting, which achieved an excellent restorative outcome in rhesus monkeys.^8^ We have also constructed a novel bioactive TEB using a 3D printed cylindrical TCP scaffold and dual-directionally differentiated hUCMSCs to repair critical-sized bone defects.^17^ The cylindrical TCP scaffolds were also adopted in the present study as a mechanical carrier of Exos. Dip-coating method was adopted to incorporate Exos into TCP, from which Exos could only be sustainably released within 48 h by a two-color near-infrared laser imaging system (data not shown) and 24 h by NTA. Since the repair of bone injury takes time, Exos must maintain an effective concentration during bone healing.^39^ To ensure a continuous therapeutic effect, local injections of Exos were carried out every three days. *In-vivo* imaging confirmed successful retention of Exos, of which the fluorescence intensity decreased from days 1 to day 2, while maintained on days 3 and 4 after additional injection on the third day. This strategy was also adopted in promoting osteochondral regeneration, of which the additional exosomes were injected every week.^40^

A critical-sized bone defect model of rats was adopted in this study, which could not completely heal by itself as was shown in the blank group. After the implantation of TCP scaffolds, a small amount of osteoid regenerated along the struts in the TCP + PBS group, indicating that the 3D printed TCP scaffold possessed good biocompatibility and excellent osteoconductive potentiality. In both TCP + PM-Exos group and TCP + OM-Exos group, newly formed bone covered the surface and the pores of the scaffolds. Specifically, the TCP + OM-Exos group bore more bone volume and mature bone quality than the TCP + PM-Exos group. This result was consistent with the *in-vitro* experiment, suggesting that the Exos secreted by osteogenically committed hUCMSCs are more powerful in promoting bone regeneration. Accumulating evidence has proved that exosomal shuttled miRNAs can posttranscriptionally regulate genes expression, thereby influencing multiple biological processes of recipient cells during bone regeneration and remodeling.^41^ miRNA sequencing was performed to compare and analyze the miRNA secretome, so as to exploit the underlying mechanism of the different pro-osteogenic properties of OM-Exos and PM-Exos.^42,43^ Compared with PM-Exos, the expression levels of miR-181a-5p and miR-100-5p were significantly upregulated in OM-Exos. Consistently, Long *et al.* find that the transcription level of miR-181a-5p is significantly increased during the osteogenic differentiation of MC3T3-E1.^44^ MiR-100-5p enhances the osteogenesis of MSCs by mTOR signaling pathway^45^ and inhibits osteoclastogenesis by modulating fibroblast growth factor 21.^46^ Meanwhile, the downregulation of hsa-miR-212-5p and miR-222-5p suggests a promoting effect of Exos in bone regeneration.^43,47^ Inhibition of miR-222 promotes chondrogenic differentiation of MSCs with higher expression of COL2A1 as well as bone healing, while overexpression of miR-222 inhibits osteogenesis-related genes expression. No studies have reported the functions of miR-4700-5p in regulating osteogenesis. We found that when being transfected with miR-4700-5p inhibitor, hBMSCs displayed a significantly higher expression of OCN, which is a representative marker of late-staged osteogenic differentiation of MSCs. A similar phenomenon was observed when hBMSCs were transfected with miR-212-5p inhibitor. The in-depth mechanisms will be elaborated in our future study. The top 10 miRNAs were enriched especially for actin filament organization and regulation of actin filament-based process. Actin is pivotal in determining the shape, spreading, and even the differentiation of cells. Osteogenically committed MSCs bear a large spreading area and high levels of actin polymerization.^48^ Furthermore, the KEGG pathway enrichment was dominated by several pathways, such as the Notch signaling pathway, which might be responsible for the pro-osteogenic activity of OM-Exos.^49^

Although the current study showed that osteogenically committed hUCMSCs-derived OM-Exos could significantly promote bone regeneration in the calvarial bone defects, there are still many obstacles from bench to bedside.^50^ At first, current isolation methods result in a low yield of Exos, which is far from satisfying the clinical demands. Therefore, the production techniques and acquisition efficiency need to be improved,^51^ such as the development of new artificial exosomes and renovation of exosomal functions.^52^ Second, it is urgent to standardize the separation, quantification, and identification of Exos.^53^ Different cell types, sources and generations, culture conditions, and isolation methods will cause large variations in the shape, size, diameter, and content of exosomes.^54^ Third, the repeat injection of exosomes will lead to a burst release pattern, which was the main limitation of this study. Therefore, a stable storage, biological safety, and reliable sustained-release system are also required to ensure sufficient retention and thus satisfactory therapeutic effects of Exos.^55^ Fourth, new techniques to accurately label and track Exos *in vivo* are of paramount importance to get the insight view of Exos.^21^ At last, it is necessary to constitute and perfect relative laws and regulations for the usage of Exos in the clinic.^56^

## IV. CONCLUSION

The present study showed both OM-Exos (from osteogenically committed hUCMSCs) and PM-Exos (from hUCMSCs) remarkably accelerated the osteogenic differentiation of hBMSCs compared with OM or PM. Specifically, OM-Exos were superior to PM-Exos in promoting osteogenesis *in vitro* and new bone formation *in vivo* when incorporated in 3D printed TCP scaffolds, which might be attributed to the differential miRNA secretome. Our study indicated that OM-Exos incorporated 3D printed TCP scaffolds hold great application potential for clinical transplantation.

## V. METHODS

### A. Characterization of the osteogenic capacity of hUCMSCs

Commercially available hUCMSCs (Cyagen Biosciences, China) were cultured in serum-free mesenchymal stem cell medium (StemRD, USA) and incubated in 5% CO_2_ at 37 °C. When cell confluence reached 80%–90%, they were digested and seeded in 48-well plates (2 × 10^4^/well). After cell adherence, the control group was cultured in DMEM (Gibco, USA) proliferation medium (PM) containing 10% FBS (Gibco, USA) and 1% penicillin-streptomycin (Gibco, USA), while the experimental group was cultured in the osteogenic induction medium (OM) including PM and osteoinductive supplements: 10 mM β-glycerophosphate (Sigma, USA), 0.2 mM ascorbic acid (Sigma, USA) and 100 nM dexamethasone (Sigma, USA). The following assays were conducted to validate whether hUCMSCs bore osteogenic differentiation properties.

#### 1. Alkaline phosphatase (ALP) activity

On days 4, 7, and 9 after induction, the cells were washed with 10% phosphate-buffered saline (PBS) and fixed with 4% paraformaldehyde (PFA) for 30 min. After that, they were incubated with the 5-bromo-4-chloro-3-indolyl phosphate/nitro blue tetrazolium (BCIP/NBT) staining reagent (Beyotime Biotechnology, China) for 20 min before being visualized by a stereomicroscope (Leica, Germany). At the same time points to ALP staining, the quantification of ALP activity was assessed. According to the manufacturer's instruction for the ALP quantitative kit (Nanjing Jiancheng Bioengineering Institute, China), the cells were lysed by 0.1% Triton X-100 and the supernatant was obtained after centrifugation. 30 *μ*l supernatant of each sample reacted with 50 *μ*l buffer solution and 50 *μ*l matrix solution at 37 °C for 15 min. Finally, 150 *μ*l color developer was added and the optical density (OD) values of the samples were immediately detected by an automatic plate reader (Thermo Scientific, USA) at 520 nm. The protein concentration of each sample was determined by the BCA protein quantitative kit (BestBio, China) for normalization.

#### 2. Alizarin red S (ARS) staining

After 14, 21, and 28 days, the cells were washed with 10% PBS before being fixed with 4% PFA for 30 min. After that, they were washed three times with ultrapure water and stained with ARS solution (0.2%, pH 8.3, Sigma, USA) at room temperature for 10–30 min. The images were captured by the stereomicroscope (Leica, Germany).

#### 3. Quantitative reverse transcription-polymerase chain reaction (qRT-PCR)

After 9 days, TRIzol^®^ (Invitrogen, USA) was used to extract total RNA. The quality and concentration of isolated RNA were evaluated using NanoPhotometer^®^ (Implen, Germany). PrimeScript^TM^ RT Master Mix (TaKaRa, China) was used for the reverse transcription. QPCR analysis was performed via TB Green^TM^ Premix Ex Taq^TM^ II (TaKaRa, China) and synthesized primers (Generay, China) in a real-time fluorescent qPCR system (Bio-Rad, USA). The expression levels of runt-related transcription factor 2 (RUNX2), collagen type 1 (COL1), and osteocalcin (OCN) were normalized to a housekeeping gene glyceraldehyde 3-phosphate dehydrogenase (GAPDH). The primer sequences are listed in supplementary material table 1.

### B. Isolation and identification of hUCMSCs-derived PM-Exos and OM-Exos

#### 1. Isolation of Exos

Serum-free mesenchymal stem cell medium was chosen as the basal medium for Exos isolation in order to avoid the influence of serum. HUCMSCs continuously cultured in the basal medium were used to harvest PM-Exos. When hUCMSCs reached 70%–80% confluence after being cultured in the basal medium for 48 h, the culture medium was changed to the basal medium with osteoinductive supplements to harvest OM-Exos. On the eighth day, the culture medium was collected and transferred into a 50 ml centrifuge tube for Exos isolation. Briefly, differential centrifugation at 4 °C was used to enrich Exos. At first, dead cells and debris were removed by centrifugation at 2000 g for 10 min. Subsequently, large vesicles and apoptotic bodies were eliminated at 10 000 g for 30 min. The remaining solution was transferred to a Beckman ultracentrifugation tube (Beckman, USA) and was centrifuged at 150 000 g for 90 min. The final precipitate was resuspended by fresh PBS in a new Beckman centrifugation tube and was centrifuged at 150 000 g for another 90 min. The sediment was carefully resuspended in 1–2 ml sterile PBS, stored at −80 °C, and termed as PM-Exos or OM-Exos.

#### 2. Identification of Exos

10 *μ*l Exos were diluted by PBS. The samples were then loaded onto a copper grid and left for 30–60 min. After being negatively stained with uranium dioxide for 5–10 min and air-dried, they were observed under a transmission electron microscope (TEM, JEOL, Japan). In addition, the size and concentration of exosomes were measured by nanoparticle tracking analysis (NTA, PMX, Germany) system. The concentration was described as particles/ml.

Western blot (WB) was thereafter applied to analyze the surface markers of Exos and host cells. RIPA containing 1% proteinase inhibitor, cocktail (ComWin Biotech, China) was used as the lysis buffer. Total protein was quantified by the BCA kit before being subjected to WB analysis. The protein (20 *μ*g) was separated by 10% SDS-PAGE gel electrophoresis, and the targeted protein was transferred to a PVDF membrane, which was blocked by 1x PBST containing 3% FBS. The primary antibody TSG101 (1:2000, Abcam, USA), Alix (1:2000, Abcam, USA), and Calreticulin (1:2000, Abcam, USA) were incubated with the membrane separately at 4 °C overnight. After incubation with the secondary antibody goat anti-rabbit (1:2000, CST), the membrane was photographed.

### C. The effects of PM-Exos and OM-Exos on the bioactivities of hBMSCs

#### 1. Uptake of PM-Exos and OM-Exos

HBMSCs were purchased from Cyagen Biosciences. To detect whether Exos could be internalized by hBMSCs, PM-Exos, and OM-Exos were labeled by Dil (Beyotime Biotechnology, China), a lipophilic fluorescent membrane dye for easy tracking. Initially, Dil was diluted to a final concentration of 10 *μ*M and incubated with Exos for 30 min at 37 °C. Exosome Spin Columns MW3000 (Thermo Fisher Scientific, USA) were used to remove the unincorporated dye. After that, labeled Exos of 10^10^ particles/ml were co-cultured with hBMSCs for 4 h. Subsequently, the supernatant was discarded and hBMSCs were washed twice with 10% PBS to remove free Exos and fixed in 4% PFA for 10 min. Hoechst 33342 (Beyotime Biotechnology, China) and Alexa Fluor^TM^ 488 phalloidin (Thermo Fisher Scientific, USA) were separately used for the staining of nuclei and F-actin. The images were obtained under a confocal laser scanning microscope (CLSM, Leica, Germany). In the blank control group, Exos were replaced by PBS to co-culture with hBMSCs.

#### 2. Transwell chemotaxis analysis

30 000 hBMSCs were seeded on the upper chambers of a Tranwell-24 plate (pore size of 8 *μ*m, Costar, Corning, USA) and cultured in DMEM containing 1% FBS and 1% penicillin-streptomycin. 500 *μ*l of DMEM containing 2 × 10^10^ particles of exosomes were added to the lower chamber. After 24 h, the non-migrated cells in the upper chamber were scraped away. The migrated cells at the bottom of the upper chamber membrane were fixed in 4% PFA. 0.1% crystal violet (Beyotime Biotechnology, China) was applied to stain the cells for 20 min. The migrated cells were captured and the cellular area was counted.

#### 3. Cell proliferation

HBMSCs were seeded in 96-well plates at a density of 3000 cells/well. The wells were randomly grouped as a complete medium group, 10^10^ particles/ml PM-Exos group, 10^11^ particles/ml PM-Exos group, 10^10^ particles/ml OM-Exos group, and 10^11^ particles/ml OM-Exos group. After the adherence of cells, the culture medium was changed and thereafter every 3 days. On days 1, 2, 3, 4, 5, and 6, cell counting kit-8 (CCK-8) was used to detect the proliferation of hBMSCs. Briefly, CCK-8 solution (1:10, Dojindo, Japan) was added into the wells and incubated at 37 °C for 2 h. Finally, the absorbance was measured at 450 nm by the automatic plate reader (Thermo Scientific, USA).

#### 4. Osteogenic differentiation

HBMSCs were seeded in 48-well plates at a density of 20 000/well for ALP and ARS staining. Culture medium containing PM-Exos or OM-Exos was applied for co-culture and changed every 3 days. The quantitative test of ALP activity was carried out on days 4 and 7 while ARS staining was performed on days 9 and 14.

To test the expression levels of osteogenesis-related genes by qRT-PCR, hBMSCs were seeded in 6-well plates at a density of 200 000/well. After incubation with PM-Exos or OM-Exos for 4 days, the expression levels of ALP, RUNX2, and COL1 were examined and normalized to GAPDH. Moreover, to find out the expression of a classical osteogenesis-related protein—BMP-2, hBMSCs were lysed to harvest the total protein after incubation with PM-Exos or OM-Exos for 7 and 10 days. The detailed experimental procedures were elaborated as aforementioned. After reacting with the primary antibody rabbit anti-human BMP-2 (1:1000, Abcam, USA) and the second antibody, the PVDF membrane was photographed and analyzed by Image J analysis software.

### D. MiRNA sequencing

Small RNAs were extracted from fresh PM-Exos and OM-Exos by exoRNeasy serum/plasma maxi kit (Qiagen, Germany). CDNA libraries were built on the basis of the QIAseq miRNA library kit (Qiagen, Germany) and subjected to miRNA sequencing with Illumina HiSeq^TM^ 3000/4000 platform at Epibiotek Co., Ltd., Guangzhou, China.

FastQC was used for quality control and raw small RNA read count matrix was obtained for the analysis of differentially expressed small RNAs by DESeq2 software with the criteria of log2 (fold change)| > 1 and FDR < 0.05. Pheatmap and ggplot2 were employed to create heatmaps and volcano plots, respectively. The enrichment analysis and functional annotation of Gene Ontology (GO) and Kyoto Encyclopedia of Genes and Genomes (KEGG) for the top 10 miRNA target genes were conducted by clusterProfiler package. The biological process (BP), cellular component (CC), and molecular function (MF), as well as the KEGG pathway, were displayed with the 10 most significant terms/pathways.

To identify potential key miRNAs that made the difference between PM-Exos and OM-Exos, miRanda (v3.3a) software program was applied to predict whether OCN was the target gene of the selected significantly expressed miRNAs. As both miR-4700-5p and miR-212-5p could bind to OCN, their mimics and inhibitors were synthesized by GenePharma, Co., Ltd., Shanghai, China. After the confluence of hBMSCs reached 60%–80%, fluorescein amidite (FAM) - labeled mimic/inhibitor negative control (NC) was separately incubated with hBMSCs according to the manufacturer's instructions. Transfection efficiency was observed by the fluorescence microscope continuously to validate the favorable transfection time. Likewise, the mimics and inhibitors of miR-4700-5p and miR-212-5p were co-cultured with hBMSCs. After 20 h, the culture medium was changed to OM. 48 h later, the cells were harvested for qRT-PCR analysis.

### E. Construction and characterization of TEB

#### 1. Fabrication and characterization of 3D printed TCP scaffolds

β-TCP (Kunshan Chinese Technology New Materials Co., Ltd.) slurry ink was prepared by dispersing β-TCP powder in distilled water with 2.5% (w/w) ammonium polymethacrylate (Darvan-C, Vanderbilt Company, Inc., USA) as a dispersant. A proper amount of hydroxypropyl methylcellulose and polyethylenimine (PEI) were successively mixed to increase viscosity and induce gelation. A bio 3D printer (Regenovo, China) was used to print the customized scaffolds with a diameter of 5 mm, a thickness of 1 mm, and a pore size of 300–400 *μ*m as previously described.^17,57^ After printing, the scaffolds were dried in air for 24 h and sintered at 1100 °C for 3 h. Before being used for cell culture and animal study, the scaffolds would be ultrasonically cleaned and sterilized.

The morphology of the scaffold was observed by scanning electron microscope (SEM, ZEISS, Germany). To confirm the cytocompatibility, 5 × 10^5^ hBMSCs were seeded on the scaffolds and cultured for 3 days. Calcein acetoxymethyl ester (AM) and propidium iodide (PI) from the live/dead viability/cytotoxicity assay kit (Beyotime Biotechnology, China) were successively co-cultured with the scaffolds for 30 min. After repeated washing, the scaffolds were observed under a fluorescence microscope (Leica, Germany).

#### 2. The release kinetics of OM-Exos

TCP scaffolds were incubated with OM-Exos (10^11^ particles/ml) solution at 37 °C for 1 h. After that, the Exos-incorporated scaffolds were bathed in 100 *μ*l PBS on a shaker at room temperature. Every 8 h, the Exos incorporated scaffolds were transferred to fresh PBS medium. NTA was used to examine the particle number of Exos.

### F. *In vivo* repair of critical-sized bone defects

With the permission of the Ethics Committee of Guangzhou Medical University (GY2019-031), 10 male Sprague–Dawley rats (6–8 weeks, weight of 150–200 g) were chosen for *in- vivo* study. The division of groups was: blank, TCP + PBS, TCP + PM-Exos, and TCP + OM-Exos (n = 5). Additional 2 rats that received DiR-labeled TCP + OM-Exos were used for *in- vivo* imaging and tracking. Briefly, DiR (Abcam, USA) was mixed with OM-Exos at a volume ratio of 1:100. After being gently pipetted and incubated for 30 min at room temperature, the un-incorporated DiR was removed by Exosome Spin Columns MW3000 at a centrifugal force of 750 g for 2 min. TCP scaffolds were incubated with DiR-labeled OM-Exos (10^11^ particles/ml) at 37 °C for 1 h.

Before surgery, the rats were fasted for 24 h. Isoflurane (Yipin, China) inhalation was used to induce anesthesia and pentobarbital sodium (50 mg/kg) (Sigma-Aldrich, USA) was used for general anesthesia by intraperitoneal injection. The operation area of the rats was shaved, sterilized, and conducted local anesthesia with 2% lidocaine. The soft tissue was cut along the middle line of the skull. The muscles and periosteum were separated bluntly to expose the bone tissue thoroughly. Two full-thickness bone defects with a diameter of 5 mm were made with a trephine under continuous saline irrigation. The scaffolds were perfectly implanted into the defects while no scaffolds were in the blank group. The wound was closed with layered sutures.

Three days post-operation, 100 *μ*l 10^11^ PM-EVs and OM-Exos particles were separately injected into the scaffolds in TCP + PM-Exos and TCP + OM-Exos groups while 100 μL PBS was injected in TCP + PBS group. The injections were conducted every 3 days continuously for 4 weeks. In the end, the rats were sacrificed by cervical dislocation under inhalation anesthesia of isoflurane, and the specimens were harvested and fixed in 4% PFA for further studies.

#### 1. *In vivo* imaging and tracking of the Exos

12 h after the operation, the rats were placed in the sample bin of a small animal *in- vivo* imaging system (PerkinElmer, USA) in a prone position. The parameters were set as an excitation wavelength of 759 nm and an emission wavelength of 779 nm. Data were obtained and analyzed on 1, 2, 3, 4, and 5 days.

#### 2. Micro-CT

Micro-CT (SkyScan 1172, Bruker, Belgium) was used for radiography analysis with the scanning parameter of 0.5-mm thickness of aluminum filter, rotation degree of 180°, pixel size of 15 *μ*m, voltage of 80 kV and current of 100 *μ*A. NRecon 1.0 software was used for reconstruction and CTvol 2.0 software for ROI selection. Threshold values were applied to select the bone tissue (80–120) and the scaffolds (140–220) in CTAn 1.13 software, so as to separately color bone tissue and the scaffolds as well as calculate bone volume/total volume (BV/TV) and bone trabecular number (Tb.N).

#### 3. Histological examination

After being decalcified with 10% EDTA for 4 weeks, the harvested samples were washed overnight, dehydrated, embedded in paraffin, and sliced at a thickness of 5–10 *μ*m. Hematoxylin & eosin (H&E) staining was applied to observe the regenerated new bone and the repair outcome under the light microscope (Leica, Germany).

### G. Statistical analysis

All the experiments were carried out at least three times. All data were analyzed by GraphPad Prism 6.0e (GraphPad Software, USA) and presented as mean ± standard deviation (SD). One-way analysis of variance (ANOVA) was used to analyze the differences between more than two groups, and Bonferroni/LSD was used for post-hoc analysis. For differences among groups at different time points, two-way ANOVA was used followed by the post-hoc test of Tukey. To test the fluorescence intensity differences of adjacent time points, a paired t-test was applied. The test level was α = 0.05, and *P* < 0.05 indicated that the difference is statistically significant.

## SUPPLEMENTARY MATERIAL

See the supplementary material for the primer sequences of qRT-PCR analysis, target gene prediction and the effects of miR-4700-5p and miR-212-5p on the osteogenic property of hBMSCs.

## Data Availability

The data that support the findings of this study are available from the corresponding authors upon reasonable request.
